# *Shewanella oneidensis* FabB: A β-ketoacyl-ACP Synthase That Works with C16:1-ACP

**DOI:** 10.3389/fmicb.2016.00327

**Published:** 2016-03-16

**Authors:** Qixia Luo, Meng Li, Huihui Fu, Qiu Meng, Haichun Gao

**Affiliations:** ^1^Institute of Microbiology and College of Life Sciences, Zhejiang UniversityHangzhou, China; ^2^State Key Laboratory for Diagnosis and Treatment of Infectious Disease, Collaborative Innovation Center for Diagnosis and Treatment of Infectious Diseases, College of Medicine, The First Affiliated Hospital, Zhejiang UniversityHangzhou, China

**Keywords:** UFA synthesis, FabB, *Shewanella*, KAS, fatty acid metabolism

## Abstract

It is established that *Escherichia coli* β-ketoacyl-ACP synthase (KAS) I (encoded by *EcfabB*) is the primary, if not exclusive, factor for elongation of the *cis*-3-decenoyl-ACP (C10:1-ACP) but not effective with C16:1- or longer-chain-ACPs. To test the extent to which these features apply to KAS I proteins in other species, in this study, we examined the physiological role of FabB in *Shewanella oneidensis*, an excellent model for researching type II fatty acid synthetic (FAS) system and its regulation. We showed that the loss of either FabA (the enzyme that introduces double bond) or FabB, in the absence of DesA which desaturizes C16 and C18 to generate respective C16:1 and C18:1, leads to a UFA auxotroph. However, fatty acid profiles of membrane phospholipid of the *fabA* and *fabB* mutants are significantly different, suggesting that FabB participates in steps beyond elongation of C10:1-ACP. Further analyses demonstrated that *S. oneidensis* FabB differs from *Ec*FabB in that (i) it is not the only enzyme capable of catalyzing elongation of the *cis*-3-decenoyl-ACP produced by FabA, (ii) it plays a critical role in elongation of C16:1- and longer-chain-ACPs, and (iii) its overproduction is detrimental.

## Introduction

**Figure 1:**
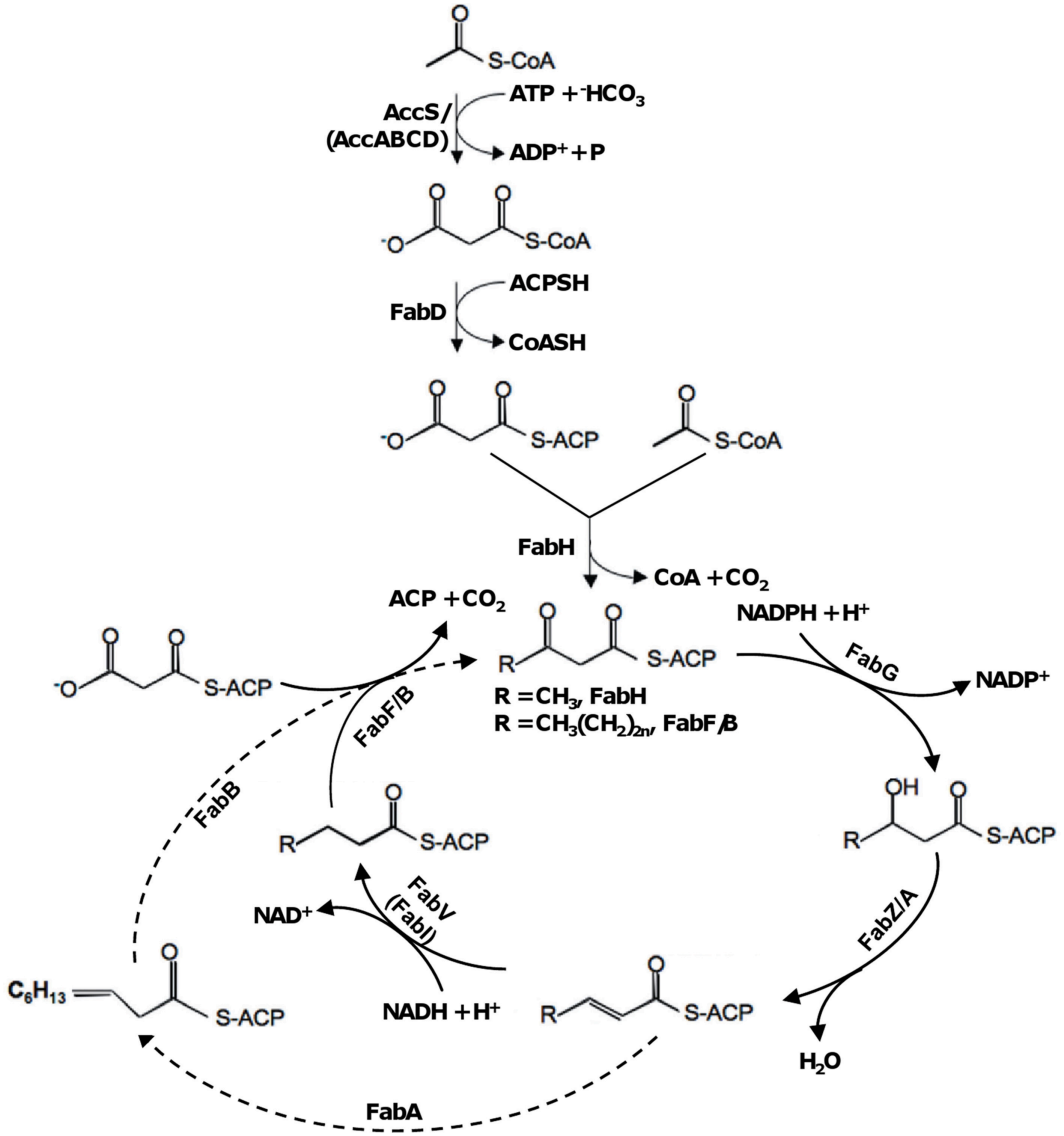
**Type II fatty acid synthesis in bacteria based on the *E. coli paradigm.*** In *S. oneidensis*, homologs of two *E. coli* enzymes, AccABCD and FabI, are not found and replaced by AccS and FabV (as in *V. cholerae* and *P. aeruginosa*), respectively. Dash line represents the steps specific to UFA synthesis.

Bacterial membrane fluidity is largely determined by the relative levels of straight-chain saturated, unsaturated fatty acids (SFAs and UFAs), and branched-chain fatty acids (BCFAs) within the phospholipids of their membrane bilayers (Campbell and Cronan, 2001; de Mendoza, 2014). The *de novo* fatty acid synthetic (FAS) pathway, named type II, is the predominant, if not exclusive, route for endogenous production of fatty acids (Parsons and Rock, 2013; Beld et al., 2015). Type II FAS pathway, the current knowledge of which derives mainly from model organism *Escherichia coli* (Cronan and Thomas, 2009), is initiated with an acetyl coenzyme A (acetyl-CoA) starter unit and a malonyl-acyl carrier protein (malonyl-ACP) extender unit (Figure 1). While acetyl-CoA is an intermediate of various metabolic pathways, malonyl-ACP is specifically produced from acetyl-CoA through two sequential steps, catalyzed by acetyl-CoA carboxylase (ACC) and malonyl-CoA-ACP transacylase encoded by *accABCD* and *fabD* respectively (Li and Cronan, 1992; Serre et al., 1995). The condensation of the starter and the extender is carried out by β-ketoacyl-ACP synthase (KAS) III (encoded by *fabH*), resulting in the butyryl thioester of ACP (Butyryl-ACP), which functions as a primer for other long-chain KAS isoenzymes (Lai and Cronan, 2003). In the cyclic pathway, the condensation of malonyl-ACP with the growing acyl chain is catalyzed by KAS I (encoded by *fabB*) and KAS II (encoded by *fabF*; (Garwin et al., 1980a; Ulrich et al., 1983; Heath and Rock, 2002).

The pathway for synthesis of all fatty acids is the same until the 10-carbon stage (C10), where it branches into the saturated and unsaturated arms. The type II FAS pathway for UFAs, also named as anaerobic pathway because oxygen is not involved, initiates with an enzyme encoded by *fabA* for introduction of the double bond at the C10 level (Figure 1). FabA catalyzes not only the dehydration of 3-hydroxydecanoyl-ACP but also the isomerization of *trans*-2-decenoyl thioester of ACP to produce *cis*-3-decenoyl-ACP (Cronan et al., 1969). Elongation of long-chain acyl-ACP relies on KAS I (FabB) and II (FabF), which control fatty acid composition and impact the rate of fatty acid production (D'Agnolo et al., 1975; Garwin et al., 1980a; Ulrich et al., 1983; Feng and Cronan, 2009). In addition to the *de novo* synthesis, UFAs can be generated by desaturation, a process distinct from the former in that oxygen is required (namely aerobic pathway; Altabe et al., 2013). To date, the aerobic pathways have been identified in many bacteria and best studied in *Bacillus subtilis* and *Pseudomonas aeruginosa* (Aguilar et al., 1998; Zhu et al., 2006). Enzymes in these pathways, which are embedded in the cytoplasmic membrane, directly desaturate the fully elongated acyl chains at different positions.

Although as KASs FabB, FabF, and FabH are structurally similar, they have differing substrate specificities and physiological functions (White et al., 2005). In *E. coli*, both FabB and FabH are essential whereas FabF is not (Cronan et al., 1969; Garwin et al., 1980a; Lai and Cronan, 2003; Feng and Cronan, 2009). Several lines of evidence suggest that FabB rather than FabH can complement the loss of FabF (Tsay et al., 1992; Jackowski et al., 2002). The failure of FabH to replace the role of FabF is due to the fact that FabH, which possesses a His-Asn-Cys catalytic triad (White et al., 2005), is active with acetyl-CoA but not with acetyl-ACP for condensation with malonyl-ACP (Jackowski and Rock, 1987; Tsay et al., 1992). In the case of FabB and FabF, although both are involved in elongation of saturated intermediates of the pathway, each catalyzes a reaction within the unsaturated branch that the other cannot (Garwin and Cronan, 1980; Garwin et al., 1980a). Unlike FabH, these two KASs have His-His-Cys active-site triads (White et al., 2005). Interestingly, overproduction of FabF but not FabB drastically affects fatty acid synthesis by blocking fatty-acid-chain elongation, leading to significant growth inhibition and viability loss (Subrahmanyam and Cronan, 1998).

*Shewanella* species, widely distributed in environments, are well known for their versatile respiration capabilities, offering great potential for bioremediation of toxic elements and serving as microbial fuel cells (Fredrickson et al., 2008; Lovley, 2012). In contrast to these beneficial roles, Shewanellae are increasingly being implicated as human pathogens in persons exposed to marine niches where most of the species thrive (Janda and Abbott, 2014). As the membrane composition of *Shewanella* is, at least in part, accountable for their widely distribution and unique physiological characteristics, we took on to investigate the fatty acid biosynthesis and its regulation in the intensively studied representative, *S. oneidensis* (Luo et al., 2014). *S. oneidensis* is of advantage for studying the subject because it is equipped not only with both anaerobic and aerobic pathways but also with FadR and FabR, the major regulators for biosynthesis and degradation of fatty acids. The presence of the aerobic pathway for UFA synthesis, which relies on a single desaturase DesA, allows mutational analysis of *fabA* and *fabB*, contrasting that *E. coli* null mutant strains lacking these genes require exogenous UFAs for growth, which prevents analyses of fatty acid composition of the membrane phospholipid (Garwin et al., 1980a; Lai and Cronan, 2003; Feng and Cronan, 2009). In addition, although *P. aeruginosa*, another well-studied model organism for the subject, possesses both anaerobic and aerobic UFA synthesis pathways, it lacks a FadR homolog. Hence, *S. oneidensis* is an ideal research model for investigating interplay of FadR and FabR in the fatty acid synthesis and regulation in bacteria equipped with both anaerobic and aerobic UFA synthesis pathways.

The essentiality of FabB for UFA synthesis in *E. coli* was established more than 4 decades ago (D'Agnolo et al., 1975). Although the enzyme catalyzes the elongation of acyl-ACPs of different chain length, except C16:1 to C18:1 (Garwin et al., 1980b; de Mendoza et al., 1983), it is now clear that the elongation of the *cis*-3-decenoyl-ACP produced by FabA is exclusively catalyzed by the enzyme (Feng and Cronan, 2009). This is achieved by utilizing a modified form of a plant thioesterase, which is active on short chain acyl-ACPs (Heath and Rock, 1995; Jones et al., 1995). Expression of the enzyme results in release of both of *cis*-5-dodcenoic acid and *cis*-7-tetracenoic acid during *de novo* fatty acid synthesis, thereby allowing identification of the step catalyzed by FabB (Feng and Cronan, 2009). As an *S. oneidensis* strain (Δ*fabA*) lacking type II UFA synthesis is able to grow without exogenous UFAs, it is conceivable that steps catalyzed by KAS I can be directly assessed by analyzing fatty acid composition. Thus, the goal of the present study was to determine the primary reactions catalyzed by KAS I, testing the extent to which the *E. coli* paradigm applies to other microorganisms. By elucidating the physiological role of FabB in *S. oneidensis*, we found that FabB functions distinctly from its *E. coli* counterpart. Most evidently, *S. oneidensis* FabB not only is able to catalyzing elongation of C16:1-ACP but also shows a detrimental effect on growth and morphology when in excess.

## Methods

### Bacterial strains, plasmids, and culture conditions

A list of all bacterial strains and plasmids used in this study are listed in Table 1 and information for primers used is available upon request. All chemicals were acquired from Sigma Co. (Shanghai, China) unless specifically noted. For genetic manipulation, *E. coli* and *S. oneidensis* strains under aerobic conditions were grown in Lysogeny broth (LB) at 37 and 30°C, respectively. When needed, the growth medium was supplemented with chemicals at the following concentrations: 2,6-diaminopimelic acid (DAP), 0.3 mM; ampicillin, 50 μg/ml; kanamycin, 50 μg/ml; and gentamycin, 15 μg/ml.

**Table 1:** **Strains and plasmids used in this study**.

**Strain or plasmid**	**Description**	**References or source**
***E. coli*** **STRAINS**		
DH5α	Host for cloning	Lab stock
WM3064	Δ*dapA*, donor strain for conjugation	W. Metcalf, UIUC
***S. oneidensis*** **STRAINS**		
MR-1	Wild type	ATCC 700550
HG1856	Δ*fabA* derived from MR-1	Luo et al., 2014
HG2774	Δ*fabF1* derived from MR-1	This study
HG3072	Δ*fabB* derived from MR-1	This study
HG4383	Δ*fabF2* derived from MR-1	This study
HG0197-1856	Δ*fabA*Δ*desA* derived from MR-1	Luo et al., 2014
HG0197-3072	Δ*fabB*Δ*desA* derived from MR-1	This study
HG2774-4383	Δ*fabF1*Δ*fabF2* derived from MR-1	This study
HG0197-2774	Δ*fabF1*Δ*desA* derived from MR-1	This study
HG0197-4383	Δ*fabF2*Δ*desA* derived from MR-1	This study
HG0197-2774-4383	Δ*fabF1*Δ*fabF2*Δ*desA* derived from MR-1	This study
HG2364-1	Δ*cco* derived from MR-1	Zhou et al., 2013
**PLASMID**		
pHGM01	Ap^r^, Gm^r^, Cm^r^, *att*-based suicide vector	Jin et al., 2013
pHG101	Km^r^, promoterless broad-host vector	Wu et al., 2011
pHG102	Km^r^, pHG101 containing the *S. oneidensis arcA* promoter	Wu et al., 2011
pHGC101	Km^r^, promoterless integrative vector	Fu et al., 2013
pHGEI01	Km^r^, integrative *lacZ* reporter vector	Fu et al., 2014
pBBR-Cre	Sp^r^, helper plasmid for antibiotic cassette removal	Fu et al., 2013
pHGE-P*tac*	Km^r^, Broad-host IPTG-inducible expression vector	Luo et al., 2013
pHGE-P*tac*TorAGFP	pHGE-P*tac* containing *gfp*	Luo et al., 2013
pHGC101-*fabB*	pHGC101 containing *S. oneidensis fabB*	This study
pHGC101-*fabF2*	pHGC101 containing *S. oneidensis fabF2*	This study
pHGEI01-P*fabB*	pHGEI01 containing the *S. oneidensis fabB* promoter	This study
pHGEI01-P*fabF1*	pHGEI01 containing the *S. oneidensis fabF1* promoter	This study
pHGEI01-P*fabG2*	pHGEI01 containing the *S. oneidensis fabG2* promoter	This study
pHGEI01-P*fabF2*	pHGEI01 containing the *S. oneidensis fabF2* promoter	This study
pPfabB-FabB-GFP	pHG101 containing P*_*fabB*_*-FabB-GFP fusion	This study
pPfabF1-FabF1-GFP	pHG101 containing P*_*fabf1*_*-FabF1-GFP fusion	This study
pPfabG2-FabF2-GFP	pHG101 containing P*_*fabG2*_*-FabF2-GFP fusion	This study
pPfabF2-FabF2-GFP	pHG101 containing P*_*fabF2*_*-FabF2-GFP fusion	This study
pParcA-FabF1-GFP	pHG102 containing FabF1-GFP fusion	This study
pHGE-P*tac*-FabA	pHGE-P*tac* containing *S. oneidensis fabA*	This study
pHGE-P*tac*-FabB	pHGE-P*tac* containing *S. oneidensis fabB*	This study
pHGE-P*tac*-EcFabB	pHGE-P*tac* containing *E. coli fabB*	This study
pHGE-P*tac*-EcFabF	pHGE-P*tac* containing *E. coli fabF*	This study

For physiological characterization, both LB and MS-defined medium containing 30 mM lactate as the electron donor were used in this study and consistent results were obtained (Shi et al., 2015). Fresh medium was inoculated with overnight cultures grown from a single colony by 1:100 dilution, and growth was determined by recording the optical density (OD) of cultures at 600 nm (OD_600_). For cultures with fatty acid additions (oleate and palmitate), which interfere with OD readings, growth was monitored by photographing colonies on plates. Mid-log-phase (~0.4 of OD_600_, unless mentioned otherwise) cells were properly diluted, plated on solid agar plates containing a paper disc of 6 mm in diameter as the size reference, and incubated at 30°C.

### In-frame deletion mutagenesis and genetic complementation

In frame deletion strains were constructed according to the *att*- based Fusion PCR method described previously (Jin et al., 2013). In brief, two fragments flanking the gene of interest were amplified with primers containing *attB* and the gene specific sequence, and then joined by a second round of PCR. The fusion fragment was introduced into pHGM01 by site-specific recombination using the BP Clonase (Invitrogen) and maintained in *E. coli* WM3064. The resulting mutagenesis vector was then transferred from *E. coli* into *S. oneidensis* by conjugation. Integration of the mutagenesis construct into the chromosome was selected by gentamycin resistance and confirmed by PCR. Verified trans-conjugants were grown in LB broth in the absence of NaCl and plated on LB supplemented with 10% sucrose. Gentamycin-sensitive and sucrose-resistant colonies were screened by PCR for the intended deletion. The deleted mutants were then verified by sequencing.

Promoterless chromosome-integrative plasmid pHGC101 was used in genetic complementation of the *fabA* and *fabB* mutants (Fu et al., 2013). A fragment containing the coding sequence and its native promoter was generated by PCR, cloned into pHGC01, and verified by sequencing. The resulting vectors were transferred to relevant *S. oneidensis* strains by conjugation, integrated into the chromosome as described before, and the antibiotic marker was then removed with helper plasmid pBBR-Cre (Fu et al., 2013).

### Expression assays

Expression of *fabB, fabF1*, and *fabF2* was assessed using an integrative *lacZ*-reporter system (Fu et al., 2014). Operon structures are referred to multiple databases (biocyc.org; microbesonline.org). Fragments of ~400 bp covering the promoter sequences were cloned into reporter vector pHGEI01 to generate transcriptional fusions. The resultant vectors were then verified by sequencing and then transferred into relevant *S. oneidensis* strains by conjugation. To eliminate the antibiotic marker, helper plasmid pBBR-Cre was transferred into the strains carrying a correctly integrated construct (Fu et al., 2013). Mid-log phase cultures grown under conditions specified in the text or figure legends were harvested, aliquotted, and subjected to β-galactosidase activity assay as described before (Fu et al., 2014).

Low expression of *fabF1* was confirmed by comparing fluorescence levels of FabF1-GFP fusion produced under the control of its native promoter and the *S. oneidensis arcA* promoter (P_*arcA*_), which is constitutively active (Gao et al., 2010). The *fabF1* gene and its native promoter was cloned into promoterless pHG101 and the *gfp* gene amplified from plasmid pHGE-P*tac*TorAGFP was placed after *fabF1*, resulting in pHG101-FabF-GFP (Wu et al., 2011; Luo et al., 2013). The coding sequence for fusion protein was amplified and placed after P_*arcA*_ within pHG102 (Wu et al., 2011). Expression of GFP fusions was visualized using a Zeiss LSM-510 confocal microscope as described previously (Luo et al., 2013).

In order to assess effect of FabA, FabB, *Ec*FabB, and *Ec*FabF of varying concentrations on growth, morphology, and fatty acid profiles, their coding genes was placed under the control of the isopropyl-β-d-thiogalactopyranoside (IPTG)-inducible P*tac* promoter within pHGE-P*tac* (Luo et al., 2013). While the P*tac* promoter within the vector in *S. oneidensis* is slightly leaky, displaying an activity of about ~50 Miller units in the absence IPTG, its strength increases proportionally with IPTG levels ranging from 0.001 to 1 mM, showing an activity of about 8000 Miller units with 1 mM IPTG (Shi et al., 2014; Chen et al., 2015; Gao et al., 2015).

### Fatty acid compositional analysis

To determine fatty acid composition, cultures of the mid-log phase grown in LB medium were collected by centrifugation, properly aliquotted, and subjected to total cellular lipid extraction as described before (Bligh and Dyer, 1959). The fatty acid methyl esters (FAMEs) were prepared by trans-esterification with 0.5 M sodium methoxide in methanol and identified using gas chromatograph-mass spectroscopy (GC-MS) (Focus GC-DSQ II) on a capillary column (30 m by 0.25 mm in diameter; Zhang et al., 2002). Helium at 1ml/min was used as the carrier gas, and the column temperature was programmed to rise by 4°C/min from 140 to 170°C, and then 3.5°C/min from 170 to 240°C for 12.5 min.

### Nadi assay

To estimate thickness of cell patches of *S. oneidensis* strains on agar plates, we took advantage of Nadi assay. Five μl of a mid-log phase culture in LB was dropped onto a LB agar plate, incubated at 30°C for 16–32 h depending on strains, and then flooded the plate with the Nadi reagents, which include α-naphthol and N',N'-dimethyl-p-phenylenediamine (DMPD; Marrs and Gest, 1973). Cell patches were timed for formation of the indophenol blue.

### Membrane integrity assay

Cells of *S. oneidensis* and *E. coli* strains grown in LB at 30 and 37°C to an OD_600_ of ~ 0.4 were adjusted to ~10^7^ cfu/ml with fresh LB, followed by four 10-fold serial dilutions. Ten microliters of each sample (from 10^4^ to 10^7^ cfu/ml) was spotted onto LB plates containing sodium dodecyl sulfate (SDS) at levels indicated in the relevant figure. The plates were incubated at 30°C before being read.

### Bioinformatics and statistical analyses

Promoter prediction for genes of interest was performed by using promoter prediction program Neural Network Promoter Prediction (Reese, 2001). Pairwise and multiple amino acid sequence alignments were performed by using Clustal Omega program (McWilliam et al., 2013). Three-dimensional (3D) structures of *S. oneidensis* FabB were predicted by using Phyre and compared to resolved structures of *E. coli* FabB (PDB accession number 2BUI; von Wettstein-Knowles et al., 2006; Kelley et al., 2015). The predicted structures were then visualized by using Pymol software (DeLano Scientific LLC). For statistical analysis, values are presented as means ± SD (standard deviation). Student's-test was performed for pairwise comparisons of groups.

## Results

### Bioinformatics analysis of fatty acid biosynthesis genes in *S. oneidensis*

As a first step of this investigation, we performed bioinformatics analysis of genes predicted to be involved in the *de novo* fatty acid synthesis in *S. oneidensis* (Figure 1 and Table 2). Apparently, the anaerobic synthesis pathway in *S. oneidensis* resembles that in *E. coli*, with most steps catalyzed by proteins highly homologous to characterized *E. coli* enzymes. Despite this, it is immediately evident that *S. oneidensis* bears new features. Firstly, the enzyme catalyzing the first committed step of the pathway is drastically different. To convert acetyl-CoA to malonyl-CoA, it is well known that prokaryotes use the ACC complex comprising four separate polypeptides, contrasting a single protein of multiple functional domains in eukaryotes (Cronan and Waldrop, 2002; Broussard et al., 2013). However, in *S. oneidensis* the Acc enzyme is encoded by a single gene, *accS* (named as Acc enzyme of a Single protein; Heidelberg et al., 2002). Interestingly, *S. oneidensis* also possesses an additional gene encoding an *E. coli* AccB homolog (Table 2). Secondly, *S. oneidensis* lacks a homolog of *E. coli* enoyl-acyl carrier protein reductase (ENR) FabI. Instead, the role is likely fulfilled by FabV, another type of ENR found in *Vibrio cholerae* and *P. aeruginosa* (Massengo-Tiassé and Cronan, 2008; Zhu et al., 2010). Thirdly, multiple candidates for FabH, FabF, and FabG are encoded in the genome (Heidelberg et al., 2002). Such a scenario is in fact rather common in bacteria; for instance, *B. subtilis* possesses two functional FabH homologs and *P. aeruginosa* uses a new class of KAS to initiate the fatty acid synthesis although it has multiple FabH orthologs (Choi et al., 2000; Yuan et al., 2012). It is therefore of interest to explore the manner through which these candidates function in *S. oneidensis*.

**Table 2:** ***S. oneidensis* homologs of *Escherichia coli* FAS II components**.

***E. coli***	**Size (a.a.)**	***S. oneidensis***	**Size (a.a.)**	***E*-value**	**Predicted function**
AccA	319	AccS (SO_0840)	1517	2e-17	acetyl-CoA carboxylase multifunctional enzyme
AccB	156			3e-08	
AccC	449			1e-84	
AccD	304			2e-06	
AccB	156	AccB (SO_0511)	152	6e-42	acetyl-CoA carboxylase biotin carboxyl carrier protein
FabD	309	FabD (SO_2777)	308	4e-143	malonyl CoA-acyl carrier protein transacylase
FabH	317	FabH1 (SO_2778)	319	6e-163	3-oxoacyl-(acyl carrier protein) synthase
		FabH2 (SO_2853)	354	2e-68	
		FabH3 (SO_2901)	341	2e-30	
FabF	413	FabF1 (SO_2774)	412	0.0	3-oxoacyl-(acyl-carrier-protein) synthase II
		FabF2 (SO_4383)	420	6e-73	
FabB	406	FabB (SO_3072)	411	0.0	3-oxoacyl-(acyl-carrier-protein) synthase I
FabG	244	FabG1 (SO_2776)	248	2e-134	3-oxoacyl-(acyl-carrier-protein) reductase
		FabG2 (SO_4382)	241	3e-56	
FabA	172	FabA (SO_1856)	171	9e-89	3-hydroxydecanoyl-(acyl-carrier-protein) dehydratase
FabZ	151	FabZ (SO_1640)	154	5e-67	(3R)-hydroxymyristoyl-(acyl-carrier-protein) dehydratase
FabV^a^	401	FabV (SO_1800)	400	0.0	enoyl-(acyl carrier protein) reductase
FabI	262	SO_2397	275	9e-14	oxidoreductase, short chain dehydrogenase/reductase family
		SO_2813	249	1e-12	
		FabG (SO_2776)	248	1e-10	3-oxoacyl-(acyl-carrier-protein) reductase

a V. cholerae FabV.

### Expression of *S. oneidensis* fabB rather than *fabFs* is affected by fatty acid species

As shown above, in *S. oneidensis* there are multiple isoforms of the KAS enzymes (Table 2). Given that all three putative FabHs (FAS III) contain a His-Asn-Cys catalytic triad and thus likely could not functionally replace FabB (KAS I) or FabF (KAS II) (Figure S1), in this study we focused on KAS I and II only. While SO_3072 is annotated as FabB (*E*-value of BLASTp to *E. coli* FabB (*Ec*FabB), e-156) there are two for FabF: SO_2774 and SO_4383 as FabF1 and FabF2 (to *Ec*FabF, e-166 and 8e-63), respectively (Heidelberg et al., 2002). Expectedly, all of these three proteins contain a His-His-Cys active-site triad (FabB, H305-H339-C169; FabF1, H305-H341-C164; FabF2, H311-H345-C174).

**Figure 2:**
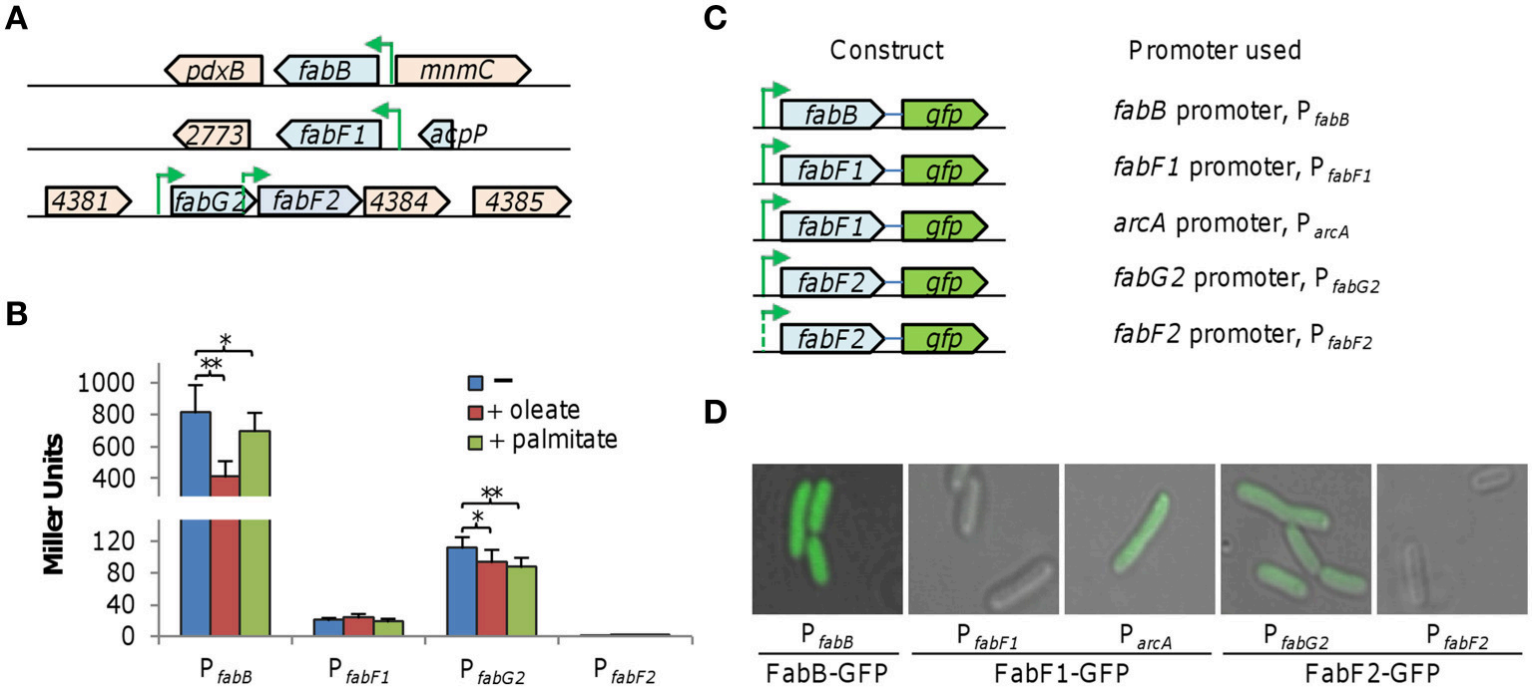
**Expression of *fabB*, *fabF1*, and *fabF2 in S. oneidensis.* (A)** Organization of the *S. oneidensis fabB, fabF1*, and *fabF2* gene regions. Operon structures are referred to multiple databases (biocyc.org; microbesonline.org). Predicted promoters P_*fabB*_, P_*fabF*1**_, P_*fabG*2**_, and P_*fabF*2**_, are shown with arrows. Genes are drawn to scale. **(B)** Influence of 0.005% oleate (C18:1Δ9) and palmitate (C16:0) on activities of the predicted promoters. Predicted promoters shown in **(A)** were cloned into an integrative *lacZ* reporter and their activity in mid-log phase cells was assayed as described in Methods. Both *fabB* and *fabF2* are responsive to fatty acid additions based on their promoter activities. Error bars represents standard deviation (SD) from at least three independent experiments. Asterisks, statistically significant difference (^*^p < 0.01; ^**^p < 0.001; *n* ≥ 3). **(C)** Constructs for GFP fusions produced under the control of indicated promoters. The *gfp* gene was placed after each gene under test and a short linker was added in between to ensure function of GFP. Each of these constructs within the same vector was introduced the wild-type. **(D)** Visualization of GFP fusions from constructs described in **(D)**. Activity of P_*fabF*1**_ is very low. Results shown are representative of three independent experiments.

To investigate the physiological roles played by these *S. oneidensis* FabB/FabF enzymes, we assessed expression of the *fabB, fabF1*, and *fabF2* genes with an integrative *lacZ*-reporter system. For genes *fabB* and *fabF1*, which are from single-gene operons (Figure 2A), fragments of ~400 bp upstream of coding sequences were amplified and cloned into the reporter vector. For *fabF2*, which is the second gene in a three-gene operon, fragments of ~400 bp upstream of its coding sequence and of the first gene (*fabG2*) of the operon were prepared. Reporter genes, without additional vector sequences, were fused in the genome as described in Methods. Cells of relevant strains grown to the mid-log phase (~0.2 of OD_600_) were collected, processed, and subjected to β-galactosidase activity assay (Figure 2B). Results demonstrated that the *fabB* promoter activity (P_*fabB*_) was ~60 and ~15 times higher than those of P_*fabF*1_ and P_*fabG*2_ (the promoter for the operon containing *fabF2*) genes, respectively. Extremely low, if not at all, β-galactosidase activity was observed from the sequence upstream of *fabF2*, suggesting a lack of an immediate promoter for this gene. When oleate (C18:1Δ9) was supplemented, activity of P_*fabB*_ exhibited a ~2-fold reduction, whereas influence of palmitate (C16:0) was much less significant. P_*fabG*2_ but not P_*fabF*1_ appeared to be modestly repressed by both UFA and SFA. Particularly, *fabF1* is found to be expressed at a very low level compared to *fabB* and *fabF2*. To confirm this, we fused GFP to the C-terminal end of these proteins and examined their production as shown in Figure 2C. Cells carrying P_*fabF*1_-*gfp* were not fluorescent and fluorescence intensities of other GFP fusions were in good accordance with data from the *lacZ* reporter. More importantly, when the DNA fragment of *fabF1-gfp* was placed under the control of *S. oneidensis arcA* promoter, which is constitutively active (Gao et al., 2010), fluorescence was visible (Figure 2D). These results, collectively, indicate that these three KAS proteins are produced at enormously different levels and production of FabB is responsive to intracellular UFA concentrations.

### Mutations in *fabA* and *fabB* cause different physiological impacts

**Figure 3:**
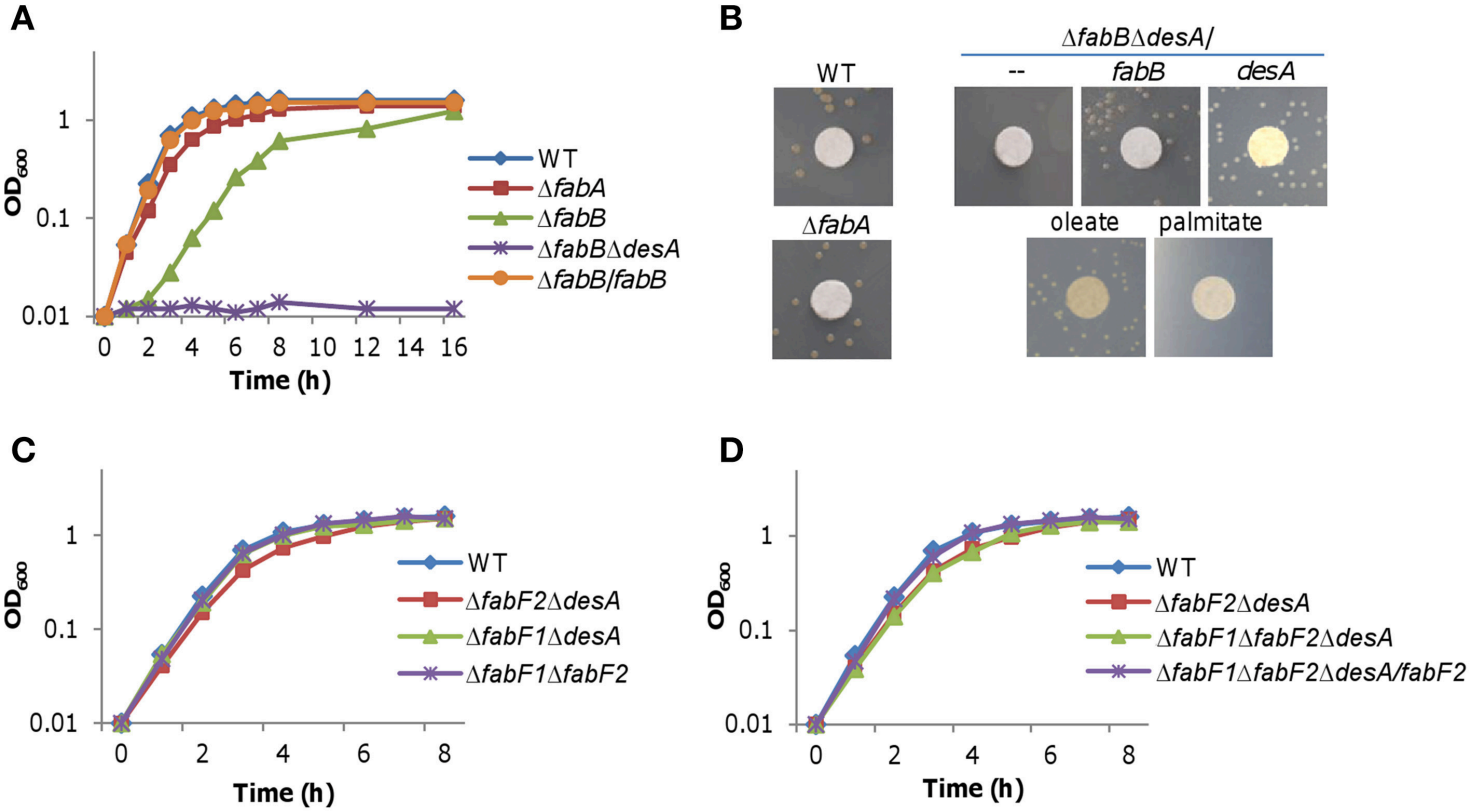
**Effects of *fabA*, *fabB*, *desA*, *fabF1*, and *fabF2*mutations on growth**. **(A)** Growth of Δ*fabA* and Δ*fabB* strains in liquid LB. Δ*fabB*/*fabB* represents the Δ*fabB* strain carrying a copy of *fabB* integrated in the chromosome driven by its native promoter. **(B)** Growth of Δ*fabB* and Δ*fabB*Δ*desA* strains on solid media under various conditions. Differences in growth were assessed with colony features: presence vs. absence, and size. A paper disc of 5 mm in diameter was placed on the plate for size assessment. Cultures of the mid-log phase for each strain were properly diluted, placed on LB plates, incubated for 24 h (for size comparison) and 48 h (for presence vs. absence) under aerobic conditions. Complementation either chemically (0.005% oleate or palmitate) or genetically (expression of *fabB* or *desA in trans*) was performed. Results shown are representative of three independent experiments. **(C,D)** Growth of Δ*fabF1*Δ*desA* and Δ*fabF2*Δ*desA* strains in liquid LB. Single mutant strains Δ*fabF1* and Δ*fabF2* were indistinguishable from the wild-type under experimental conditions (not shown). Δ*fabF1*Δ*fabF2*Δ*desA*/*fabF2* represents the triple mutant carrying a copy of *fabF2* integrated in the chromosome driven by its native promoter. In **(A–C)**, error bars (less than 10% of the average), representing SD from three independent experiments, were omitted for clarity.

Although FabA is essential to anaerobic UFA biosynthesis in *S. oneidensis*, its loss impedes aerobic growth only modestly (~15% reduction by generation time; Luo et al., 2014; Figure 3A), suggesting that UFAs generated by desaturase DesA are nearly sufficient to support normal growth. If *S. oneidensis* FabB, resembling its *E. coli* counterpart, primarily catalyzed elongation of the *cis*-3-decenoyl-ACP produced by FabA, we would expect that its loss results in a similar defect in growth. To test this, we constructed a *fabB* in-frame deletion mutant for *S. oneidensis*. In liquid medium under either aerobic or anaerobic condition, the Δ*fabB* strain grew significantly slower, approximately 58% relative to the wild-type based on generation times (Figure 3A). We then examined effect of exogenous fatty acids on growth defect of the strain lacking *fabB*, which was performed on plates because of their interference with OD readings (Figure 3B). In the absence of DesA, the defect resulting from the *fabB* mutation (Δ*fabB*Δ*desA*) was partially recoverable by oleate but not by palmitate, suggesting that impaired UFA production caused by the loss of the *fabB* gene is vital to survival and growth. The defect can be attributed to the intended mutation because it was corrected by expressing a single copy of the *fabB* gene *in trans*. Consistently, the depletion of FabB retarded growth more substantially than the FabA loss, as illustrated in the Δ*fabB*Δ*desA* strain expressing a copy of *desA* (Figure 3B). While these data indicate that both FabA and FabB catalyze a step in UFA synthesis that cannot be conducted by other enzymes, significant difference in growth resulting from their loss manifests that FabA and FabB impact on the *S. oneidensis* physiology distinctly.

To test whether FabB can functionally complement the loss of FabF, we created strains lacking *fabF1, fabF2*, or both in *desA*^+^ and *desA*^−^ backgrounds. All resulting mutants, Δ*fabF1*, Δ*fabF2*, Δ*fabF1*Δ*fabF2*, Δ*fabF1*Δ*desA*, Δ*fabF2*Δ*desA*, and Δ*fabF1*Δ*fabF2*Δ*desA*, were able to grow without exogenous UFAs (Figures 3C,D). In addition, there was no difference in growth observed between the wild-type and strains lacking *fabF1, fabF2*, or both (Figure 3C). These results illustrate that FabB can fulfill the role played by two FabF proteins for UFA production because FabB is the only possible candidate encoded in the genome for elongation of long-chain acyl-ACPs in the *fabF1fabF2* mutant. However, although Δ*fabF1*Δ*desA* grew indistinguishable from the wild-type, growth of the Δ*fabF2*Δ*desA* was slightly but significantly slower (Figure 3C), implicating that FabF2 is necessary, but FabF1 is dispensable, for optimal UFA/SFA production, at least under experimental conditions. The additional removal of the *fabF1* gene from the Δ*fabF2*Δ*desA* strain did not further worsen growth (Figure 3D), supporting that the growth defect is attributed to the loss of FabF2. This is then confirmed by successful complementation of the Δ*fabF1*Δ*fabF2*Δ*desA* strain with a copy of *fabF2* integrated in the chromosome (Figure 3D). Thus, these data collectively indicate that although FabB can act as a functional replacement for FabF2 it appears less effective for the steps catalyzed preferably by the latter *in vivo*.

### Loss of fabB but not fabA significantly alters colony morphology

**Figure 4:**
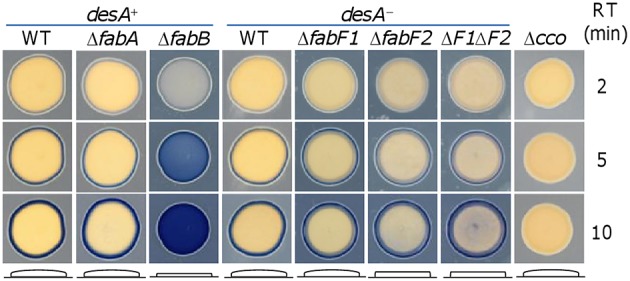
**Effects of *fabA*, *fabB*, *fabF1*, and *fabF2* mutations on morphology**. Morphology of cell patches grown from a drop of mid-log phase culture for each strain on LB plates was assessed by Nadi assay as described in Methods. The blue ring formed by Nadi assay manifests the activity of cytochrome *c* oxidase, which is missing in the negative control Δ*cco*. Morphology of cell patches for each strain is illustrated by respective diagram given at the bottom. Nadi reagents could penetrate through the boundary of cell patches that are too thin as seen with Δ*fabB*, Δ*fabF2*, and Δ*fabF1*Δ*fabF2*. Three reaction times (RT) were presented to show the progress of the Nadi reaction. Δ*F1*Δ*F2* represents Δ*fabF1*Δ*fabF2*. The diameters of the patches were 8 ± 1 mm. Presented are representative results of three independent experiments.

Besides the growth difference in liquid media resulting from *fabA* and *fabB* mutations, we noticed that colonies from the *fabB* mutant on LB plates were considerably thinner than those of the wild-type and Δ*fabA* strains. To better illustrate this phenotype, cell patches developed from a culture drop of each strain were used instead of colonies since the *fabB* mutant could not grow to suitable size for the analysis (Figure 4). We then took advantage of the Nadi assay, which is based on the rapid formation of indophenols blue from colorless α-naphtol catalyzed by cytochrome *c* oxidase with an exogenous electron donor (Marrs and Gest, 1973). Although the assay is initially designed to specifically detect activity of cytochrome *c* oxidases, we found that it is excellent in distinguishing the thickness difference of cell patches. As expected, the strain (Δ*cco*) lacking the cytochrome *cbb*_3_ oxidase, which is the only functional cytochrome *c* oxidase in *S. oneidensis* (Zhou et al., 2013; Yin et al., 2015), gave a negative result. In contrast, the blue rings surrounding cell patches of the wild-type and *fabA, fabB*, and *desA* single mutant strains appeared at similar rates, illustrating that activity of the cytochrome *cbb*_3_ oxidase is not affected by these mutations. However, Δ*fabB* differed from other three strains in that its cell patches became blue entirely with time. This is likely due to the reduced thickness, which permits the Nadi reagents to diffuse into cell patches over the boundary. We then examined effect of FabF1 and FabF2 on the morphology in the absence of DesA. Consistent with growth phenotype, the loss of FabF1 had no impact on the thickness of cell patches (Figure 4). However, the strain lacking FabF2 or both FabF2 and FabF1 formed patches modestly thinner, albeit not comparable to that of Δ*fabB*. These results indicate that the *fabB* mutation prevents cells from developing a community impermeable to the Nadi reagents whereas FabF proteins are not critical to this process in general.

Another possible explanation to the Nadi-penetrating phenotype is increased membrane permeability of individual cells, which could not be arbitrarily excluded as membrane properties are probably altered when FabB is absent. To test this notion, we assessed sensitivity of the Δ*fabB* strain to SDS, which disrupts membranes (Seddon et al., 2004). However, as shown in (Figure S2) the wild-type and Δ*fabB* strains had similar sensitivity to SDS whereas the negative control (Δ*arcA*) was hypersensitive (Wan et al., 2015). Similar results were obtained from the Δ*fabF1*Δ*fabF2* strain. All of these data indicate that the loss of FabB may not significantly alter membrane properties with respect to membrane integrity although it is essential for forming cell patches with normal morphology.

### fabB in excess is detrimental

**Figure 5:**
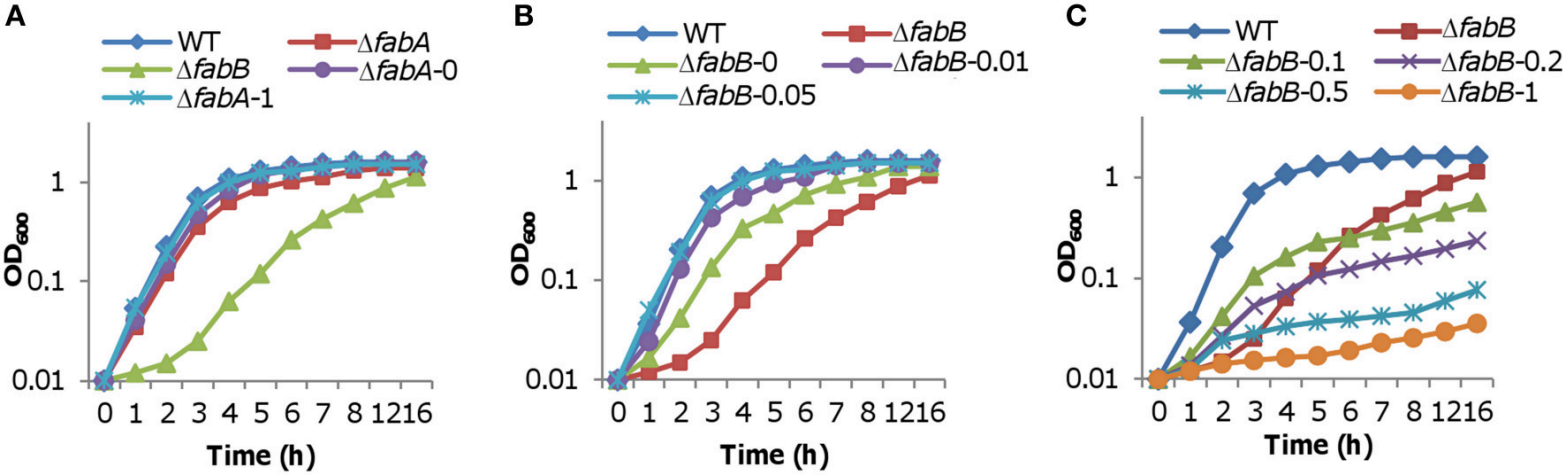
**Influence of FabA and FabB overproduction on growth**. Expression of *fabA* and *fabB* at varying levels was achieved by using the IPTG-inducible P_*tac*_ within pHGE-P*tac*, which is slightly leaky in *S. oneidensis*. The vector with the *fabA* and *fabB* construction was introduced in the respective deletion strains by conjugation. **(A)** Influence of FabA overproduction on growth. Experiments were conducted in the absence and presence of IPTG from 0.05 to 1 mM (given after strain name). Given that growth of Δ*fabA* and the wild-type with all levels of IPTG was indistinguishable, results with 1 mM were shown only. **(B,C)** Influence of FabB overproduction on growth. Experiments were conducted in the absence and presence of IPTG from 0.01 to 1 mM (given after strain name). In all panels, error bars (less than 10% of the average), representing SD from three independent experiments, were omitted for clarity.

To gain more clues for unraveling the mechanisms underlying the defects resulting from the loss of FabB, we examined effects of FabA and FabB in overabundance on growth. Vector used for this purpose was pHGE-P*tac*, in which genes of interest is under the control of the IPTG-inducible promoter P_*tac*_ (Luo et al., 2013). Our previous studies have revealed that the promoter, which is slightly leaky, drives expression proportionally with IPTG levels up to 1 mM (Shi et al., 2014; Chen et al., 2015; Gao et al., 2015). For the assay, *fabA* and *fabB* genes were cloned into the vector individually and the resultants were introduced to the respective mutant strains. Expression of the *fabA* gene in the presence of IPTG ranging from 0.05 to 1 mM eliminated the growth difference between the wild-type and Δ*fabA* strains, indicating that *S. oneidensis* is amiable to FabA levels (Figure 5A). In the case of FabB, a different pattern was observed. In the absence of IPTG, significant growth restoration was observed because of the leakiness of the vector (Figure 5B). When IPTG was added to final concentration of 0.05 mM, full complementation of growth defect due to the *fabB* mutation was achieved (Figure 5B). However, expression of the *fabB* gene induced by IPTG at 0.1 mM hampered growth significantly (Figure 5C). Given that IPTG at higher concentrations further deteriorated growth, these data conclude that FabB functions in a dose-dependent manner in *S. oneidensis*.

**Figure 6:**
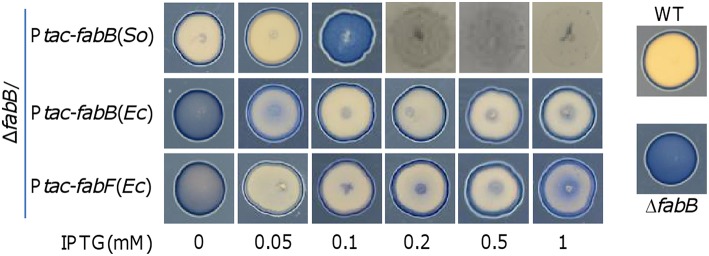
**Effect of *S. oneidensis FabB* as well as *E. coli* FabB and FabF at varying levels on morphology of *S. oneidensis* cell patches**. Experiment was performed the same as described in Figure 4. Production of indicated proteins in the Δ*fabB* strain was controlled by IPTG. Experiments were conducted independently at least three times and the representative was presented.

To test whether FabB in excess affects morphology of cell patches on plates as in its absence, we conducted the Nadi assay (Figure 6). Consistent with growth data, expression of *fabB* without IPTG significantly improved the morphology and with 0.05 mM IPTG fully corrected the morphology defect. However, in the presence of 0.1 mM IPTG the cell patches resembled those from the *fabB* mutant, implying that the function of FabB at this level is balanced for its detrimental effect with respect to this phenotype. Despite this, it should be noted that the impacts on physiology resulting from the FabB loss and FabB overproduction by 0.1 mM IPTG are different, evidenced by their distinct growth curves: slow growth rate vs. dramatically reduced biomass (Figure 5C). Further increased FabB production by IPTG as low as at 0.2 mM nearly abolished growth on plates (Figure 6), indicating that *S. oneidensis* is highly sensitive to overabundant FabB.

### Loss of fabB results in accumulation of C14 fatty acids

**Figure 7:**
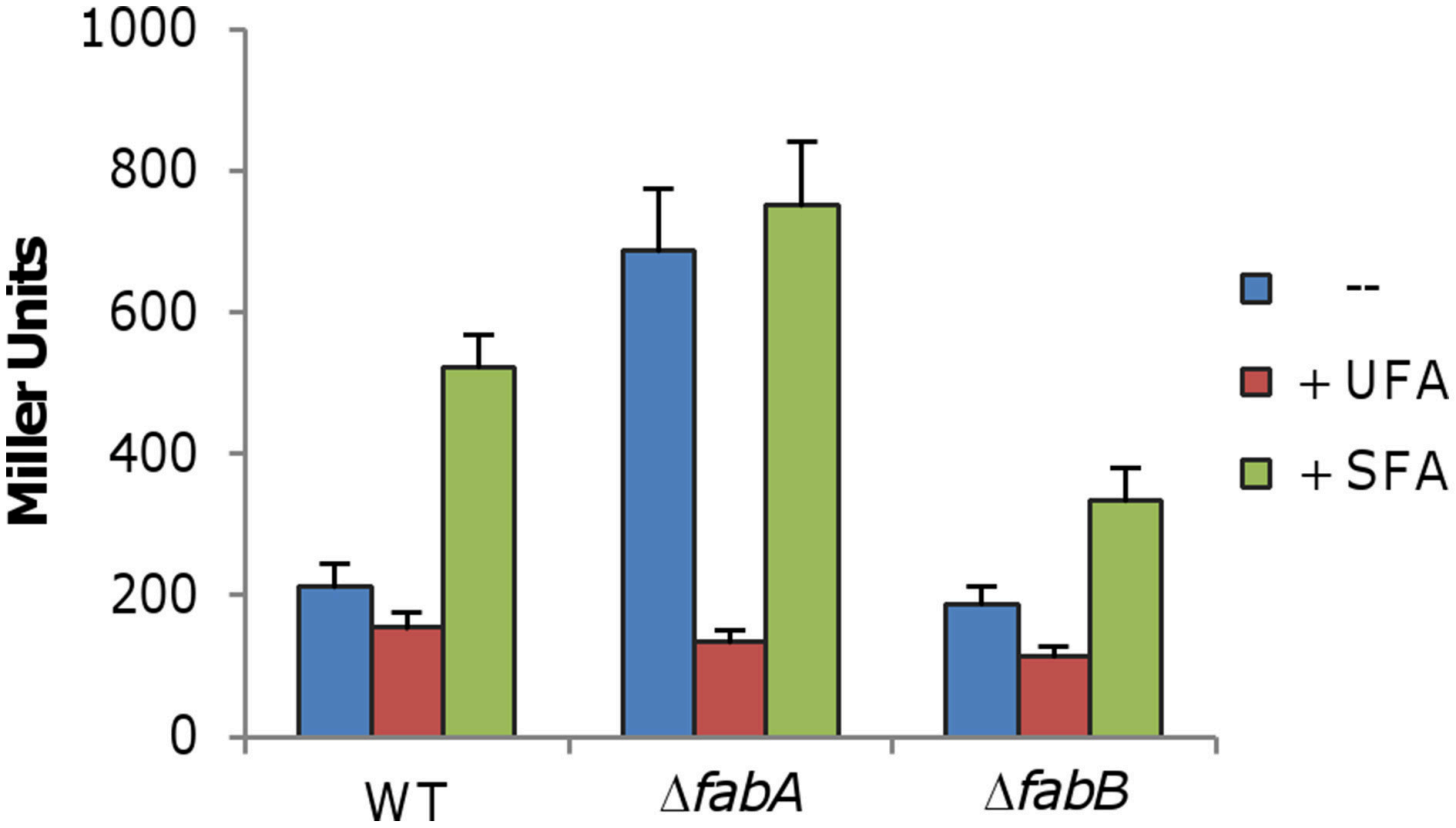
**Expression of *desA* under various conditions**. The *desA* promoter was cloned into an integrative *lacZ* reporter, integrated into the chromosome of the relevant strains, and assayed the same as Figure 2. Effects of *fabA* and *fabB* mutations, in combination with exogenous UFA (C18:1Δ9) and SFA (C16:0), were assessed. Experiments were conducted independently at least three times and standard deviations were presented as error bars.

To explore the mechanisms underlying the phenotype resulting from the *fabB* mutation, we intended to compare the fatty acid profiles of the Δ*fabA* and Δ*fabB* strains. In the absence of FabA, the *desA* gene is up-regulated more than 3-fold to compensate for the loss of the primary source of UFAs via FAS (Luo et al., 2014). Hence, we first determined whether the *desA* gene is also expressed differently when FabB is depleted. The integrative P_*desA*_-*lacZ* reporter was introduced into the *fabB* mutant and β-galactosidase activity was assayed in cells prepared the same as for the *fabA* mutant (Luo et al., 2014). Surprisingly, expression of the *desA* gene appeared to be unaffected by the *fabB* deletion, in contrast to the *fabA* mutation (Figure 7). These data implicate that the UFA production defect resulting from the FabB loss may not be compensated by increased production of DesA.

As expected, we found that the fatty acid profiles of the Δ*fabA* and Δ*fabB* strains varied substantially (Table 3). Consistent with previous report (Luo et al., 2014), the *fabA* mutant showed an overall similar fatty acid profile as the wild-type, although modest impacts on C14:0 and C18:0 fatty acids were observed. Notably, loss of FabA showed negligible influence on levels of UFAs. In contrast, depletion of FabB affected UFA species drastically, reducing the percentage of UFAs from 35% for the wild-type to 19% for Δ*fabB*. While both C16:1 and C18:1 decreased up to ~3-fold, C14:1 showed a ~6-fold increase. More strikingly, the abundance of C14:0 increased ~14 times and C16:0 coincidently reduced to 55% relative to the wild-type level. As C16 and C18 species dominate the fatty acid composition in mesophilic bacteria, the drastic enhancement in C14 species in the *fabB* mutant may account for its growth and morphology defects. If this is the case, we reason that FabB in excess would reduce C14 levels. Indeed, there were barely C14 species in Δ*fabB* cells producing FabB by 0.1 mM IPTG (Table 3). On the contrary, the levels of C18:1 rose substantially, approximately 4 times relative to those in the wild-type. Meanwhile, C16:1 remained at low levels, similar to those in the Δ*fabB* strain. In the case of FabA in overabundance, the fatty acid composition was found to be similar to that of the wild-type, consistent with the finding that FabA in excess does not exert noticeable impacts on physiology. These results suggest that FabB is important for conversion of C14:1 to C16:1 and then C18:1.

**Table 3:** **Fatty acid composition of *S. oneidensis* strains^a^**.

**Strain**	**C14:1**	**C14:0**	**C15:0**	**C16:1**	**C16:0**	**C18:1**	**C18:0**
WT	1.36 ± 0.22	1.79 ± 0.34	26.33 ± 4.74	27.52 ± 2.62	26.45 ± 3.64	6.59 ± 0.64	8.58 ± 1.06
Δ*fabA*	0.65 ± 0.31	5.55 ± 0.32	28.15 ± 3.45	24.58 ± 3.57	23.19 ± 4.61	5.05 ± 0.43	5.54 ± 0.53
Δ*fabB*	8.75 ± 1.02	23.34 ± 4.35	28.27 ± 4.00	9.12 ± 1.38	14.58 ± 3.46	1.26 ± 0.29	9.13 ± 1.27
Δ*fabA*/*SofabA*^b^	2.03 ± 0.45	2.26 ± 0.57	24.55 ± 3.81	29.51 ± 6.37	27.15 ± 4.43	6.16 ± 1.23	7.71 ± 1.17
Δ*fabB*/*SofabB*^b^	0.69 ± 0.12	–	21.52 ± 3.37	12.69 ± 2.28	27.53 ± 4.35	22.48 ± 3.29	17.33 ± 2.42
Δ*fabB*/*EcfabB*^c^	3.57 ± 0.58	14.84 ± 1.69	29.63 ± 4.56	33.51 ± 4.15	34.73 ± 4.11	1.38 ± 0.23	3.49 ± 0.49
Δ*fabB*/*EcfabF*^c^	–	0.77 ± 0.56	21.84 ± 3.78	32.39 ± 4.33	25.21 ± 3.69	14.55 ± 2.82	9.72 ± 1.55

a Data were presented in percentage of the total fatty acids.

b Expression of SofabA and SofabB were controlled by IPTG at 0.1 mM.

c Expression of EcfabB and EcfabF were controlled by IPTG at 0.2 mM.

### Complementation of the *S. oneidensis fabB* mutant by *E. coli fabB* and *fabF*

**Figure 8:**
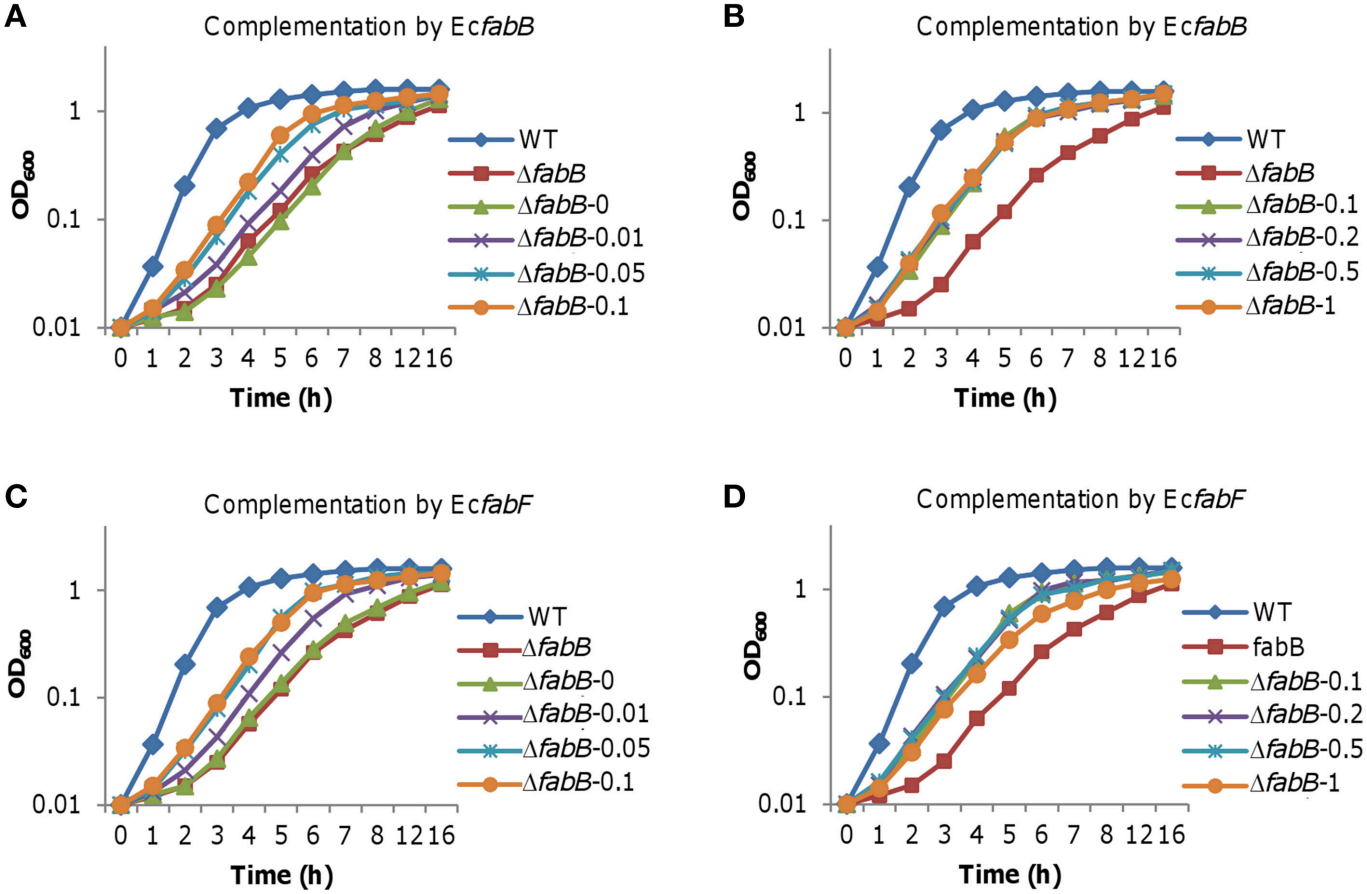
**Effect of *E. coli* FabB and FabF at varying levels on growth of *S. oneidensis***. Growth of the Δ*fabB* strain carrying *EcfabB* and *EcfabF* driven by P_*tac*_ in liquid LB was monitored. IPTG at indicated concentrations was used to control production of *E. coli* FabB and FabF. **(A,B)** the Δ*fabB* strain carrying *EcfabB*. **(C,D)** the Δ*fabB* strain carrying *EcfabF*. In all panels, error bars (less than 10% of the average), representing SD from three independent experiments, were omitted for clarity.

Unlike FabB of *S. oneidensis*, overproduction of *E. coli* FabB (*Ec*FabB) does not significantly harm cells but plays a beneficial role in defending antibiotics that targets KASs (de Mendoza et al., 1983; Jackowski et al., 2002). We therefore hypothesize that *Ec*FabB may complement the defect of *S. oneidensis* due to FabB depletion but not impede growth when in overabundance. *EcfabB* was cloned and placed under the control of P_*tac*_ and the resulting vector was introduced into the Δ*fabB* strain as described for the *S. oneidensis* counterpart. By comparing to *S. oneidensis* FabB (Figure 5), *Ec*FabB produced without IPTG had no effect in complementing the mutation (Figure 8A). On the contrary, in the presence of IPTG as low as at 0.01 mM, *Ec*FabB was able to significantly promote growth of the Δ*fabB* strain (Figure 8A). When IPTG was added to no more than 0.1 mM, growth improvement increased with IPTG concentrations. However, IPTG at higher concentrations up to 1 mM did not further facilitate growth (Figure 8B). Importantly, *Ec*FabB produced by IPTG at 1 mM did not show any negative effect on growth. We then examined the effect of *Ec*FabB on *S. oneidensis* Δ*fabB* cells grown on plates (Figure 6). Consistently, *Ec*FabB produced from the leaky promoter did not evidently improve the morphology (thickness of cell patches). When IPTG was added to 0.05 to 1 mM, the Δ*fabB* strain developed much thicker cell patches, with 0.1 mM showing best result, which excellently matches the growth data presented in Figure 8. These results collectively manifest that *Ec*FabB in excess does not exert detrimental impacts in *S. oneidensis*, implicating that there is a significant difference in functions between these two highly homologous FabB proteins. To address the difference, we performed GC-MS analysis of membrane fatty acid composition of the Δ*fabB* strain expressing *EcfabB* with 0.2 mM IPTG (Table 3). Although *Ec*FabB reduced levels of the C14 species significantly (compared to the Δ*fabB* strain without *Ec*FabB), these fatty acids were considerably more abundant than those in the wild-type. Moreover, cells had higher levels of C16 species, in contrast to C18 species with *S. oneidensis* FabB. These data suggest that *Ec*FabB is much less effective than *S. oneidensis* FabB in catalyzing elongation of C14 to C16 species and C16 to C18 species.

We then assessed effect of *E. coli* FabF (*Ec*FabF) on the Δ*fabB* strain when expressed to various levels. The effect of *Ec*FabF produced without IPTG on growth of the *S. oneidensis* Δ*fabB* strain, albeit rather limited, was evident (Figure 8C). Similar levels of complementation were achieved with IPTG at 0.05 to 0.5 mM, being significantly better than with IPTG at either 0.01 or 1 mM. As *Ec*FabF produced in the 1 mM retarded growth slightly, it implies that *Ec*FabF in excess has a negative influence. This observation was supported by growth results on plates (Figure 6). In the case of fatty acid composition, *Ec*FabF produced in the presence of 0.2 mM IPTG eliminated the C14 accumulation resulting from the *fabB* mutation (Table 3). However, there was notable increase in the amount of C18:1 species. These data suggest that the compromised complementation by *Ec*FabF in excess may be due to the accumulation of long chain fatty acids, a scenario observed with *S. oneidensis* FabB in overabundance.

## Discussion

In bacterial type II FAS pathway, the chain extension steps to form the carbon-carbon bond via Claisen condensation are catalyzed by KASs, all of which are derived from a thiolase precursor enzyme (Jiang et al., 2008). KAS III (FabH), the enzyme responsible for the initiation of fatty acid synthesis, is highly active on substrates with less than four carbons but inactive with acyl-ACPs (Tsay et al., 1992). Hence, the extension cycle is driven by two other KASs, KAS I (FabB) and KAS II (FabF; Garwin et al., 1980a; Ulrich et al., 1983). In *E. coli*, FabB rather than FabF is essential, and its loss results in a UFA auxotroph (Cronan et al., 1969; Lai and Cronan, 2003). This is due to that the elongation of the *cis*-3-decenoyl-ACP produced by FabA depends on FabB predominantly, if not exclusively, and thus the activity of FabB is the primary factor in determining cellular UFA content (Feng and Cronan, 2009). While these findings lay a profound foundation to the current understanding of the type II FAS system in bacteria, in this study we presented evidence that there exist great variations in function of KAS I proteins.

Due to the presence of desaturase DesA, *S. oneidensis* mutants losing ability to synthesize UFAs via the type II FAS pathway can be readily obtained and conveniently studied (Luo et al., 2014). In *S. oneidensis*, the loss of either FabA or FabB leads to a UFA auxotroph in the absence of DesA. However, the *fabA* and *fabB* single mutants are distinct from each other in growth; the latter has a growth defect considerably more severe than the former. Cells without FabA could not produce *cis*-3-decenoyl-ACP, the very first substrate for UFA production via the type II FAS pathway, thus leading to the complete loss of the UFA production via the type II FAS pathway. Surprisingly, FabA depletion does not cause significant changes in fatty acid composition (Table 3). This is likely due to increased production of DesA because the contents of UFAs in the *fabB* mutant, in which the amount of DesA is not affected, are significantly lower than those in the wild type. Thus, the growth defect resulting from the FabA loss may be due to relatively low effectiveness of DesA on UFA generation. It is worth mentioning that a *P. aeruginosa* strain lacking FabA displays substantial reduction in growth rate (estimated to be ~35% reduction, vs. ~15% in *S. oneidensis*) although the microorganism possesses two desaturases for aerobic UFA production (Zhu et al., 2006). Presumably, contribution of aerobic pathways to overall UFA production is likely to vary greatly among microorganisms.

To explain severe growth defect of the *fabB* mutant, we speculate that the FabB loss may alter amounts of certain intermediate fatty acids formed through the pathway, which in turn worsens growth. This appears to be the case. The fatty acid composition of the strain devoid of FabB differs from those of the wild-type and *fabA* mutant strains substantially (Table 3). While further in-depth exploration is needed, one likely explanation is that a significant portion of *cis*-3-decenoyl-ACP is still routed into the UFA synthesis branch in the absence of FabB. Our data clearly support this notion as the *fabB* mutant produces a large amount of C14:1. The C14:1 species could not be attributed to DesA because desaturases of this type only work with C16 and C18 fatty acids (Zhu et al., 2006). The loss of FabB leads to drastically elevated levels of C14 fatty acids and coincident reduction in the abundance of C16 and C18 fatty acids, including both the saturated and the unsaturated. This gains support from overexpression of FabB, which results in enhanced production of C18 fatty acids. Thus, it appears that C14- and C16-ACP rather than C10- and C12-ACP are good substances for *S. oneidensis* FabB, with C14-ACP as the best. In the context of C16-ACP, this feature of *S. oneidensis* FabB is in contrast to *E. coli* FabB, which is poor in elongating C16-ACP (Garwin et al., 1980b; de Mendoza et al., 1983). Given that the effect of *Ec*FabB overproduction on the abundance of C18 in *S. oneidensis* is considerably minor compared to its *S. oneidensis* counterpart, we conclude that *S. oneidensis* FabB and *Ec*FabB differ from each other in that the former is much more effective than the latter in catalyzing elongation of C16 to C18 species. The functional difference between these two FabB proteins is further demonstrated by the partial complementation. Unlike *S. oneidensis* FabB, *Ec*FabB produced at various levels fails to fully correct defects in either growth or morphology of the *S. oneidensis fabB* mutant. We do not yet know the mechanism underlying the difference. Predicted *S. oneidensis* FabB structure by computational modeling proves to be very similar to *Ec*FabB (Figure S3), suggesting that sequence and structural variations accountable for functional distinction between these two proteins are rather subtle.

In *E. coli*, elongation of C16 to 18 relies on FabF (Garwin and Cronan, 1980; Garwin et al., 1980a). In addition, EcFabF in excess results in lethality of *E. coli* cells (Subrahmanyam and Cronan, 1998), an effect that is observed from *S. oneidensis* FabB. Despite this, *S. oneidensis* FabB is not a functional counterpart of *Ec*FabF with respect to their physiological roles because the latter when produced at varying levels is unable to fully rescue the defect caused by the former. This is likely due to the fact that *Ec*FabF cannot catalyze the elongation of *cis*-3-decenoyl-ACP (Garwin and Cronan, 1980; Garwin et al., 1980a). To date, FabF proteins that can function as a replacement for *Ec*FabB have been reported, but exclusively in bacteria lacking a homolog of *Ec*FabB, including *Lactococcus lactis, Enterococcus faecalis*, and *Clostridium acetobutylicium* (Wang and Cronan, 2004; Morgan-Kiss and Cronan, 2008; Zhu et al., 2009). Based on that the *S. oneidensis fabB* mutant is still able to proceed to C14:1, it is almost certain that there must be a protein(s) other than FabB that can catalyze the elongation of *cis*-3-decenoyl-ACP. In this sense, *S. oneidensis* presents a novel model for bacteria having FabB.

Unlike FabB, which alone is sufficient to carry out reactions for generation of fatty acids that are required for survival and growth, two homologs (FabF1 and FabF2) of *Ec*FabF are dispensable. Hence, in *S. oneidensis* FabB functionally overlaps all roles played by FabF proteins but not vice versa, as in *E. coli* (Cronan et al., 1969; Lai and Cronan, 2003). We propose that one of FabFs may be able to carry out the elongation of *cis*-3-decenoyl-ACP. Intriguingly, although our data suggest that *S. oneidensis* FabF2 is probably the functional homolog of *Ec*FabF, *fabF1* is the one that not only has higher sequence similarity but also share the same synteny with *EcfabF*. We speculate that the insignificance of FabF1 in physiology, if not lost completely, is likely due to its extremely low expression. Efforts to test this hypothesis are under way.

Many questions regarding roles of KAS I and II proteins remain to be addressed. *S. oneidensis* FabB seems to work effectively in SFA synthesis as its loss and overproduction affect both saturated and unsaturated fatty acids. It is therefore unexpected that the growth defect of the *fabB* mutant is recovered in the presence of exogenous oleate but not palmitate. In addition, mechanisms for functional differences between KAS I and II proteins remain elusive. Some findings suggest that their capabilities of interacting with FadD, the malonyl-CoA:ACP transacylase which converts malonyl-CoA to malonyl-ACP, may have a role (Garwin et al., 1980a; Magnuson et al., 1992; Serre et al., 1995). The lethality caused by *Ec*FabF overproduction is proposed to be associated with accumulation of malonyl-CoA, a result of reduced activity of *Ec*FabD (malonyl-CoA:ACP transacylase) due to interaction with *Ec*FabF (Subrahmanyam and Cronan, 1998). While all KASs could form a complex with FabD as suggested in *E. coli* (Garwin et al., 1980a), it is possible that their capacities for the interaction vary significantly. Furthermore, these observations presented here, along with reported before (Morgan-Kiss and Cronan, 2008) imply a fine line between KAS I and II proteins with respect to their physiological functions, which is surely supported by high similarities in their sequences and structures (White et al., 2005).

## Author contributions

HG conceived the idea and designed the project. QL, ML, HF, and QM carried out the experiments. QL, ML, HF, and HG analyzed data. QL, ML, and HG wrote the paper.

### Conflict of interest statement

The authors declare that the research was conducted in the absence of any commercial or financial relationships that could be construed as a potential conflict of interest.

## Abbreviations

ACP: acyl carrier protein
CoA: coenzyme A
FAS: fatty acid synthesis/synthetic
KAS: β-ketoacyl-ACP synthase
BCFA: branched-chain fatty acid
SFA: saturated fatty acid
UFA: unsaturated fatty acid.
