# Supplementary material for: Shewanella oneidensis FabB: A β-ketoacyl-ACP Synthase That Works with C16:1-ACP
Source: Front Microbiol. 2016 Mar 16;7:327. doi: 10.3389/fmicb.2016.00327 (PMC4793157; doi:10.3389/fmicb.2016.00327)
Supplement: Supplementary file 1 [file Presentation1.PDF]

Supplemental materials of

Shewanella oneidensis FabB: a β-ketoacyl-ACP synthase that works with C16:1-ACP

Qixia Luo,[1,2#] Meng Li,[1#] Huihui Fu[1], Qiu Meng[1], and Haichun Gao[1]*

[1]Institute of Microbiology and College of Life Sciences, Zhejiang University, Hangzhou, Zhejiang, 310058, China

[2]Collaborative Innovation Center for Diagnosis and Treatment of Infectious Diseases, State Key Laboratory for Diagnosis and Treatment of Infectious Disease, The First Affiliated Hospital, College of Medicine, Zhejiang University, Hangzhou, Zhejiang 310003, China

[#]These authors contributed equally to this work.

```
FabH3(SO2901)      MKQVVISGSGLFTPPHGISNEALVESFNAYVDMFNLENAGLIEQGHVEALSYSSCEFIEK
FabH2(SO2853)      MQYATITGWGKCVPPATLTNDDLAT------------------------FIETSDEWIKS
FabH1(SO2778)      -MHTKILGTGSYVPVQVRSNQDLEK------------------------MVETSDQWIVE
EcFabH             -MYTKIIGTGSYLPEQVRTNADLEK------------------------MVDTSDEWIVT
                      . * * *   *    :*  *                          :  :* ::*

FabH3(SO2901)      ASGIKHRYVMVKEGILDPEVMMPLIPERSSDELSMQAEIGVEAALMALKQAELKAEQIDL
FabH2(SO2853)      RTGISQRHISHV--------------------NT--SELASVAAKRALAAAGIEGSEIDM
FabH1(SO2778)      RTGISERRIAAQ--------------------DETVSTMGYLAALNALEMAGIEASDLDM
EcFabH             RTGIRERHIAAP--------------------NETVSTMGFEAATRAIEMAGIEKDQIGL
                    :** .* :                           : :.  **  *:  * :: .:: :

FabH3(SO2901)      VIVACAYTQRAYPAMAIEIQRALGTRG-YGYDMQVACSSATFAIVAAANAIATGSASRVL
FabH2(SO2853)      IILASASPDTLIPNIASTVQANIGA-NCAAFDINAACSGFLYGLGLASSQIKSGQCKKVL
FabH1(SO2778)      IICGTTSAANAFPAAACEIQAMLGVHTIPAFDIAAACSGFVYALSVADQFVKNGTAKKVL
EcFabH             IVVATTSATHAFPSAACQIQSMLGIKGCPAFDVAAACAGFTYALSVADQYVKSGAVKYAL
                   :: . :      *  *  :*  :*     .:*: .**:.  :.:  * . : .*  . .*

FabH3(SO2901)      VINPEICSAQVNYRDRDSHFIFGDVATAVVLEEQSLVAPNKGFTILSSRCFTDYSNNIRS
FabH2(SO2853)      VVGAERLSFYLDWSRRETAVLFGDGAGAVVVEATDIPGGVLGYELNNDP-----DGRDIL
FabH1(SO2778)      VIGADVLSRLCEPEDRTTIILFGDGAGAAIIGASDEPGIIS-THIYADG-----RQGDLL
EcFabH             VVGSDVLARTCDPTDRGTIIIFGDGAGAAVLAASEEPGIIS-THLHADG-----SYGELL
                   *:  :  :   :   * : .:*** * *.::   .  .      :  .

FabH3(SO2901)      NFGFLNRCDPSSAHQADKLFHQQGRKVFKELLPMIYQHLDEHLAEQSLTPQSFKRLWLHQ
FabH2(SO2853)      KAGFGTAMDRFSADSLDFYIQFDGQEIFKRAINGMNKLSTQVLEKCGVDKDEVDLVIPHQ
FabH1(SO2778)      KCAFPPRQGE--TSEAVGFMTMKGNDVFKVAVTQLSHVVTETLRLNNIDKSEIDWLVPHQ
EcFabH             TLPNAD--RV--NPENSIHLTMAGNEVFKVAVTELAHIVDETLAANNLDRSQLDWLVPHQ
                   .             .    :   *..:**  :  : :   : *    :  .... :  **

FabH3(SO2901)      ANINMNLFVVKKLLGDEVAPEQAPVVLDEYANTASAGSVIAFHQY--SADFNAGDLGLLS
FabH2(SO2853)      ANERIIDTLVSRM---KIPKEKAFVNIANYGNTSAATIPIAICDALEKGLIKPNQTILSC
FabH1(SO2778)      ANFRIINATAKKL---DMSLDKVVLTLAKHGNTSAASVPIALDEAVRDGRIQRGQLLLLE
EcFabH             ANLRIISATAKKL---GMSMDNVVVTLDRHGNTSAASVPCALDEAVRDGRIKPGQLVLLE
                   ** .:    ..::    :  ::. : : .:.**::*    *: :   .. ::  :  *

FabH3(SO2901)      SFGAGYSIGSVILQKR---------------------------------
FabH2(SO2853)      AFGAGLTSAALLLQWGERVTPVQISDAQLPPCDQSGIELVKRAVDYFCK
FabH1(SO2778)      AFGAGFAWGSALVRF----------------------------------
EcFabH             AFGGGFTWGSALVRF----------------------------------
                   :**.* : .: :::
```

Figure S1. Sequence alignment of *Ec*FabH and *S. oneidensis* putative FabH proteins. Residues that constitute the His-Asn-Cys catalytic triad are highlighted (Cys112, His244, and Asn274 in *Ec*FabH). The alignment was performed by using Clustal Omega.

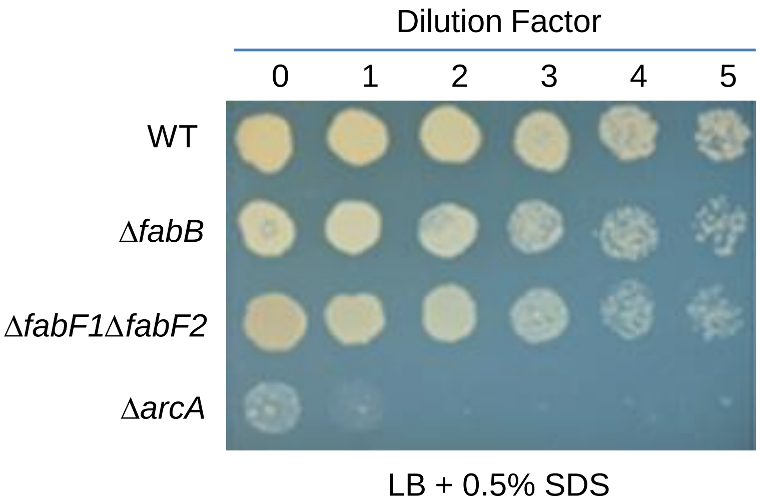

Figure S2. Loss of *fabB* or both *fabF1* and *fabF2* does not affect susceptibility to SDS*.* The culture of ~10^8 cells/ml was decimally diluted and 5 µl of each dilution was dropped onto LB plates containing 0.5% SDS. The plates were incubated at 30ºC for 24 h before photograph was taken. A strain lacking ArcA (**a**erobic **r**espiration **c**ontrol protein, a global regulator), which is hypersensitive to SDS, was used as the negative control. The assays were conducted at least three times and representative results were presented.

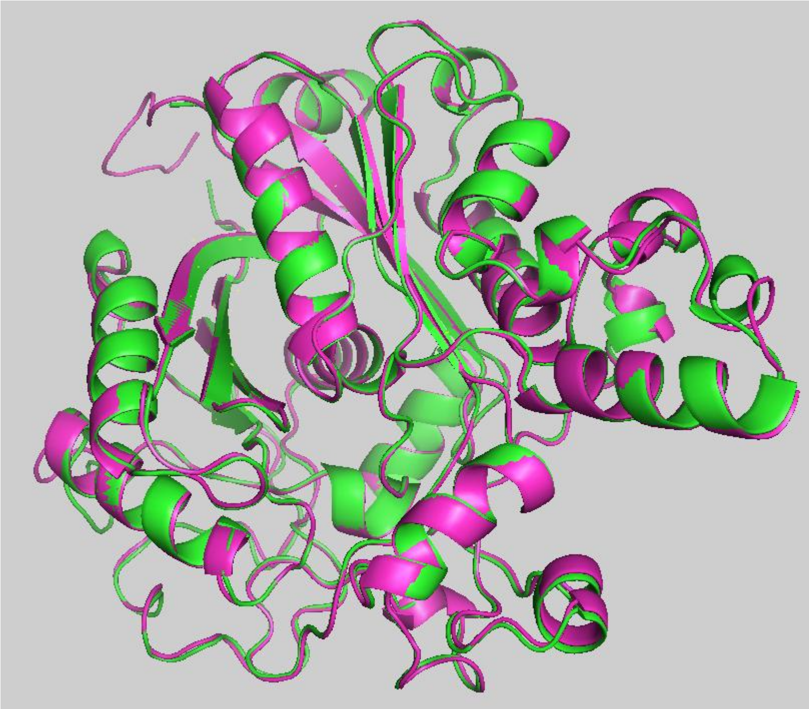

Figure S3. Structural comparison of *S. oneidensis* and *E. coli* FabB. Three-dimensional (3D) structures of *S. oneidensis* FabB were predicted by using Phyre and compared to resolved structures of *E. coli* FabB (PDB accession number 2BUI). Shown is a superimposition of *S. oneidensis* FabB (green) and *E. coli* FabB (purple).
